# Immunometabolism in cancer: a systemic perspective

**DOI:** 10.3389/fimmu.2025.1656776

**Published:** 2025-10-08

**Authors:** Helena Moretti, Giorgia Cioccoloni, Esperanza Perucha

**Affiliations:** ^1^ Centre for Inflammation Biology and Cancer Immunology, Department of Inflammation Biology, King’s College London & Centre for Rheumatic Diseases, King’s College London, London, United Kingdom; ^2^ School of Food Science & Nutrition, University of Leeds, Leeds, United Kingdom

**Keywords:** cancer, systemic immunometabolism, immunotherapy, obesity, T cells

## Abstract

Cancer incidence is increasing, becoming a significant public health concern. Cancer arises from the uncontrolled division of cells that cannot be restrained by the anti-tumour response mounted by the immune system. Both tumour and immune cells require high levels of energy in the form of ATP and synthesis of macromolecules to support differentiation and proliferation. To support these metabolic demands, adaptations at the cell, tissue and systemic level are required. Here, we take a systemic perspective to summarise the energetic needs of the anti-tumour response and how metabolic overload and obesity affects these processes. We describe how immunotherapies that aim to reverse immune cell exhaustion have unexpected effects depending on the metabolic background of the patient and finally we propose the use of this knowledge to advance current cancer prevention and treatment strategies.

## 1 Introduction

Cancer will affect 1 in 2 people in the UK according to Cancer Research UK, with newly diagnosed cases mostly present in people aged 75 or over, and a higher prevalence in individuals from white ethnic backgrounds (1). Cancer is considered a complex systemic disease, where the interplay between genetic and environmental factors drives the transformation of normal cells (2). Once tumorigenesis is achieved, it is the role of the immune system to detect these abnormalities and mount an efficient anti-tumour response. When these responses fail, cancer develops and can spread to other tissues, leading to loss of tissue homeostasis and clinical symptoms.

Research efforts to develop better treatments mean that cancer survival has doubled in the last 50 years (1). Among these treatments, immunotherapy – aimed at improving the ability of the immune system to recognise and destroy malignant cells – has proven key in this success, with checkpoint blockade, adoptive cell therapy and vaccination at the forefront of current improvements in survival rates (3). However, not all patients benefit or respond to treatments, and recurrence is also possible, creating a real need for novel approaches towards improved therapy and prevention.

Both cancer and immune cells are dependent on cellular and systemic metabolism to proliferate and differentiate. Taking cancer as a systemic disease, here we explore the relationship between metabolism, immunity and cancer at the whole individual level. We also address this relationship under metabolic overload settings, such obesity, and explore how systemic metabolic dysregulation affects the immune response to cancer and the efficacy of anticancer immunotherapies such as checkpoint inhibitors. With this knowledge, we propose targeting systemic metabolism for improving cancer treatment, either on its own or as adjuvant into current immunotherapies. While exploring these avenues, we have also found several gaps in knowledge that might inspire future work on this important societal topic.

## 2 Systemic immunometabolism

**Figure 1:**
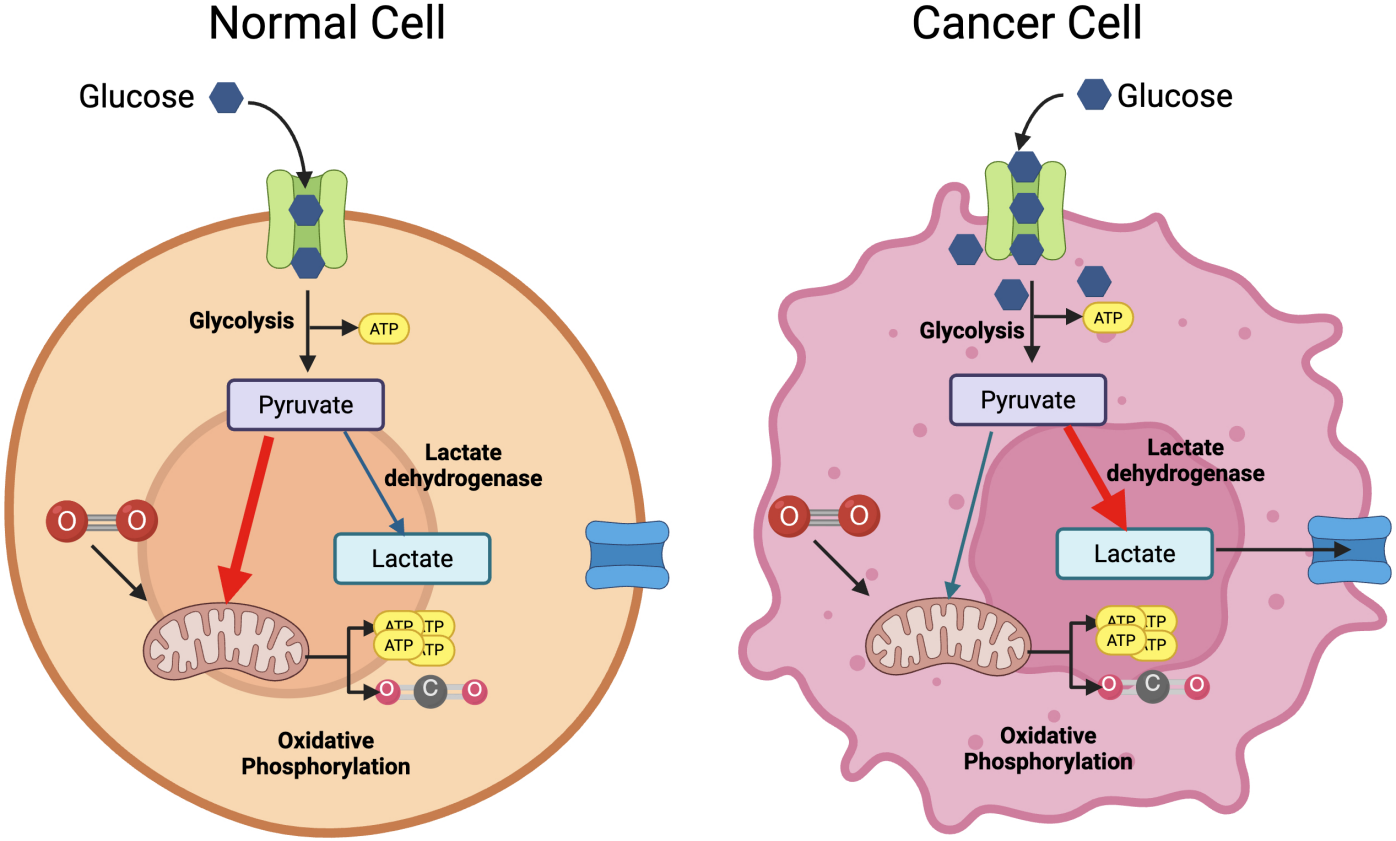
The Warburg effect. Under normoxia, cells will metabolise glucose into pyruvate to feed oxidative phosphorylation and generate substantial amounts of ATP (left panel). Cancer cells convert glucose into pyruvate through aerobic glycolysis, that generates some ATP and intermediates for biosynthetic pathways (right panel). This same effect is seen in resting vs.activated T cells. Created with BioRender.

From the birth of the immunometabolism field, much of the research efforts have been focused on understanding how cellular metabolic pathways and nutrient sensing and uptake impact on the outcome of the immune response – the so-called cellular metabolism. From these studies, we have learnt that metabolic reprogramming underlies the development of an adequate immune response, from the differentiation of naïve cells to the formation of immune memory or the resolution of the immune response. Cellular immunometabolism has been excellently reviewed elsewhere (4, 5) so we will not cover this topic in depth here. However, it is important to highlight that highly proliferative cells, like cancer cells and immune cells, undergo metabolic reprograming when activated, switching from oxidative phosphorylation to glycolysis even in the presence of oxygen (**Figure 1**). This phenomenon – the so-called Warburg effect - was first described in the 1920s by Otto Warburg (6). It presented a paradox at the time, as oxidative phosphorylation is a much more efficient metabolic route to obtain the energy required for proliferation and differentiation. We now know that active cells rely on glucose metabolised through glycolysis not only to generate ATP, but also to generate substrates for biosynthesis of macromolecules that support cell division and function.

The immune system, and immune cells, are like no other system in the body. Not only they are present in every tissue of the human body, but they are able to adapt to very different environments in terms of nutrient and oxygen availability, circulating from blood to tissues, where they have a critical role in maintaining tissue homeostasis and reacting to danger. This implies that cellular metabolic reprogramming must be influenced by the systemic metabolism and vice versa, and this relation is important to maintain a healthy body. However, the relationship between systemic metabolism and immunity – systemic immunometabolism – is much less understood. On one hand, systemic metabolism controls nutrient and growth factor availability, which immune cells sense and require to become activated and mount an appropriate immune response. On the other hand, the immune response against pathogens or cancer is a highly demanding process, both in terms of anabolism (requiring proliferation and biosynthesis of effector molecules) and catabolism (30% of total body energetics upon infection) (7). From this, one can assume that whole body energetics must either sense inflammation and/or can be controlled by the immune system at a certain level.

### 2.1 Metaflammation

The first evidence of the relationship between an active immune response and systemic metabolic alterations dates from 1883-1884, when the association between *meningococci meningitis* infection and transient diabetic syndrome was described (8). These observations pointed at increased insulin resistance upon infection, that was further confirmed in both animal models (9) and acute infection in humans (10). In addition to this, evidence of the presence of immune infiltrates in metabolic tissue was already known, although a possible functional role of the infiltrate was not interrogated (11, 12). The cellular and molecular mechanism underlaying all these observations remained unknown until landmark works from Feingold and Lang described how Tumour Necrosis Factor alpha (TNFa), a potent pro-inflammatory cytokine, induced insulin resistance *in vivo* (13, 14). Further studies described increased levels of TNFa in obese adipose tissue in both animal models and humans, while TNFa neutralisation led to improvement in metabolic parameters such insulin resistance in mice (15–17). However, even up to this day, a causal relationship between inflammation and systemic metabolic changes is still not fully described in humans (18, 19). Despite this, the presence of an abundant immune cell resident population in metabolic tissue suggests their importance in maintaining tissue homeostasis as well as performing surveillance functions. Moreover, resident cells are key players in the mechanism of action of current immunotherapies, as we will discuss later.

**Figure 2:**
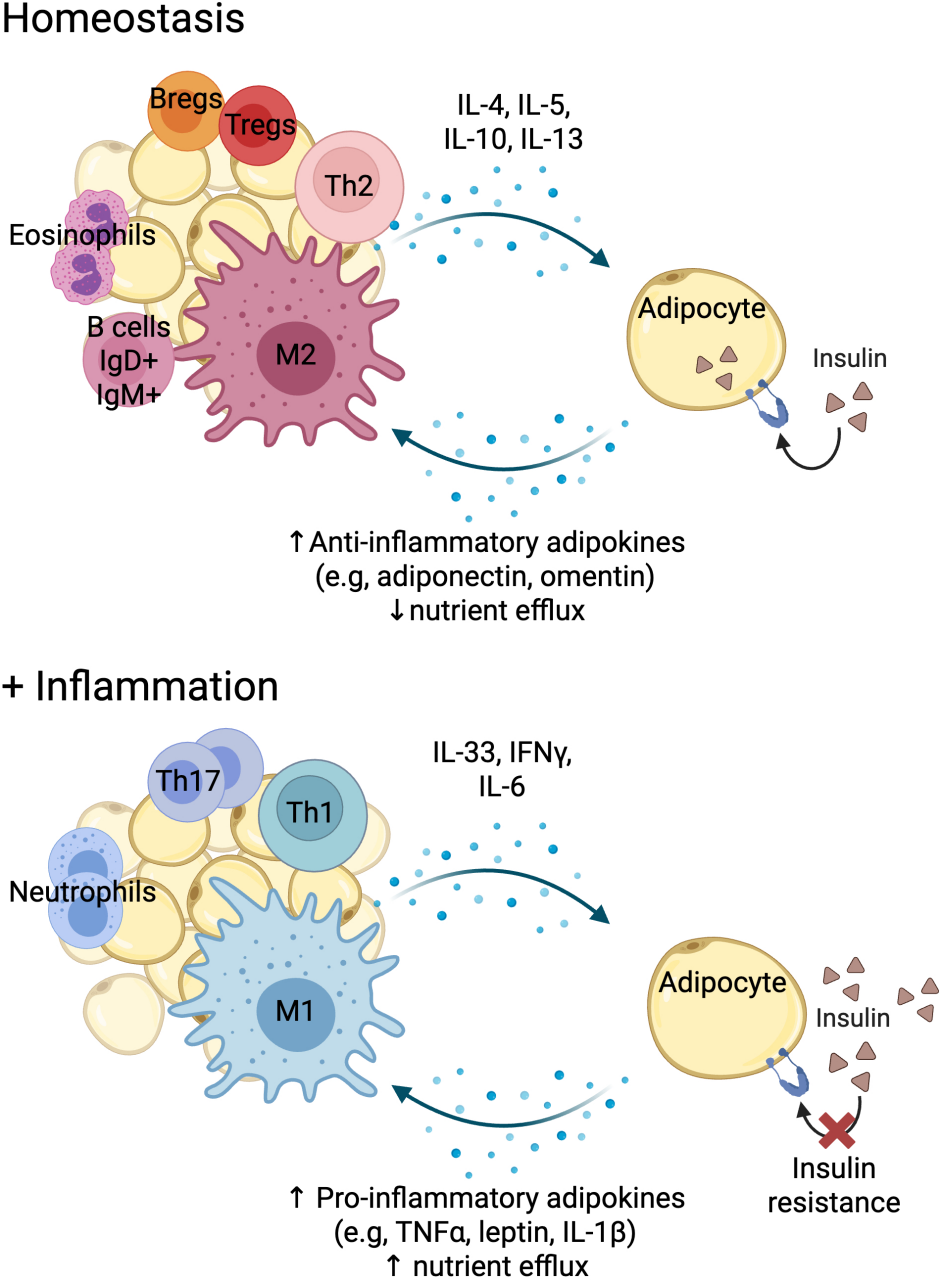
Immune cell-adipose tissue crosstalk in homeostasis and inflammation. Created with BioRender.

At the tissue level and in homeostatic conditions, this is, in the absence of any danger, stress or inflammation, the metabolic tissue immune infiltrate is predominantly of regulatory nature, with abundant regulatory T cells (Tregs) and homeostatic macrophages (M2 type) that promote correct insulin signalling in non-immune cells such adipocytes or hepatocytes (20, 21). Immune cells adapt to their environment by even acquiring the expression of master transcription factors of metabolic cell function, like Peroxisome proliferator-activated receptor gamma (PPARγ) (22) (**Figure 2**). This cellular landscape changes upon immunological challenge, like an infection. Infection by most pathogens with high replicative capacity such as virus and bacteria, requires a type 1 response, associated with T cell activation and the production of the cytokines interferon gamma (IFNγ) and TNFα, that will activate innate immune cells like macrophages (M1 type) to target the pathogen (**Figure 2**). This type of response also requires a high proliferative rate, which is supported by glucose. In order to shift nutrient allocation towards the immune system, a transient state of insulin resistance is induced in metabolic tissue (23). This molecular crosstalk is mediated by pro-inflammatory cytokines and adipokines.

A very similar landscape is observed upon metabolic overload, where increased inflammation in metabolic tissue has been widely described. Akin to infection, the immune infiltration consists of T cells producing IFNγ (T-helper cells type 1, Th1) and interleukin (IL)-17 (T-helper cell type 17, Th17) pro-inflammatory cytokines and M1 macrophages, establishing a feedback loop where low grade chronic inflammation induced by nutrient excess leads to chronic insulin resistance in metabolic tissue (18, 19, 24, 25). Interestingly, nutrient excess in an aberrant environmental condition that is exclusive to humans. Moreover, it is a recent event in evolutionary terms, hence there has been no requirement for survival pressure to evolve mechanism to protect the body from caloric excess (as opposed to food deprivation) and as such there are limited natural options to return the body to homeostasis (18, 26).

Metabolic overload in metabolic tissue is generally the consequence of “Western lifestyle” patterns, linked to high caloric intake, lack of physical exercise and other factors that overstrain systemic metabolism. A key manifestation of metabolic overload is obesity, that is associated with reduced immune function. Obese individuals have increased susceptibility to infections and reduced vaccine responses - a similar phenotype observed in aging - possibly due to continuous immune activation over time (27). These alterations include a reduced function in primary and secondary lymphoid organs (28–31)*;* and increased proportions of dysfunctional immune cells (29, 32–34).

### 2.2 Cancer and systemic immunometabolism

The progression from tumoral transformation of normal cells into clinically relevant cancer depends on one hand on the capability of tumour cells to proliferate and migrate to other tissues and on the other hand, the ability of the immune system to mount an adequate anti-tumour response. Both processes depend on metabolic cues. As already explained, cancer and immune cells undergo metabolic reprograming, a requirement for their high proliferative rate and acquisition of effector function. As such, numerous cancer-related genetic mutations or tumour-suppressor genes have a direct effect on cellular metabolism, highlighting how important metabolism is as a target when thinking about cancer treatment or even prevention (35). Additionally, many cancers develop adjacent to adipose tissue, and they can manipulate adipocyte biology to provide metabolites that fuel cancer cell division, growth and metastasis. Moreover, cancer cells can also induce a dedifferentiation programme in the adipocytes that further support these processes (36, 37). As a consequence, calorie restriction had been proposed to inhibit cancer growth for the last 100 years (38, 39).

If cancer and metabolism are deeply interlinked, then it is no surprise to observe that obesity has profound effects on cancer risk and development. At the population level, a “Western lifestyle” – associated with a high diet caloric intake – is widening across Asian and African countries and this has been associated with increased rates of cancer in these countries. Similarly, migration to a “Western lifestyle” country is accompanied by the acquisition of the indigenous population’s cancer risk (40–42). These epidemiological observations suggest that, at least in these cases, cancer should be preventable with interventions that target the “Western lifestyle”. This is a changing paradigm in the current cancer clinical arena, as described in this excellent review by Holly et al. (43).

Overall, obesity has been associated with both cancer risk and progression (36, 44). The mechanism(s) that link both are intense areas of research and implicate immune (chronic inflammation, increased systemic pro-inflammatory cytokines), metabolic (hyperinsulinemia, dysregulation of leptin and adiponectin levels) and endocrine (steroid hormones) factors. Moreover, many of the therapies used to treat cancer today specifically target the immune system, seeking to harness and amplify the body’s built-in anti-tumour response. Given the profound effect of obesity on the immune system and cancer risk, it is necessary to consider how obesity affects the efficacy and mechanism of these immunotherapeutic approaches. To explore this topic further, we will first consider mechanistic insights into the phenotypic and functional changes that occur in T cells, in the context of both cancer and obesity.

## 3 Mechanistic insights

### 3.1 T cell exhaustion in obesity and cancer

T cell exhaustion is a dysfunctional cell state, classically described as arising due to chronic antigenic stimulation (45), but hypoxia and glucose deprivation have also been shown to have important roles in driving the exhaustion process (46, 47). Exhaustion is characterised by increased expression of immune inhibitory receptors, weakened effector function, reduced self-renewal capacity, altered epigenetics, and a specific transcriptional programme and metabolism (48). T cell exhaustion is one of the major causes leading to immune escape of cancer, creating an environment that supports tumour development and metastatic spread (49). In addition, T cell exhaustion plays a pivotal role in the efficacy of current immunotherapies for cancer, hence we will next provide a comprehensive view of the role of T cell exhaustion in cancer development and progression.

The key drivers of exhaustion are present in most cancers. Tumour infiltrating lymphocytes will be consistently stimulated by tumour antigen presented by antigen-presenting cells, often over a prolonged period prior to diagnosis. Tumours are also highly hypoxic tissues which consume considerable amounts of nutrients compared to healthy tissues, so T cells in the vicinity of tumours will also experience hypoxia and nutrient deprivation (50). It has been found that tumour-infiltrating lymphocytes in patients with a range of cancers have high proportions of exhausted T cells (49, 51).

Interestingly, several studies also demonstrate an increase in T cell exhaustion as a result of obesity, possibly due to the continuous immune activation present in both conditions. In obese mouse models, T cell exhaustion is described not only in adipose tissue T cells (52), but also in peripheral blood, liver and spleen, particularly when combined with aging (34, 53). Though there is no definitive answer yet as to the exact mechanisms of obesity-driven T cell exhaustion, there is evidence that adipocytes in the obese state can upregulate major histocompatibility complex class II (MHC-II) and act as antigen-presenting cells to adipose tissue infiltrating T cells (54). Furthermore, excess adiposity is linked to increased leptin secretion (**Figure 2**). Leptin is a hormone involved in regulating appetite and energy storage, but it has also been shown to affect T cell differentiation pathways. T cells (especially CD4^+^) exposed to leptin are more likely to differentiate into a pro-inflammatory phenotype, increasing production of pro-inflammatory cytokines (54). This pro-inflammatory adipose environment, with increased levels of cytokines such as TNFα and IL-6 (55) along with chronic T cell stimulation through MHC-II leads to T cell exhaustion.

Rather than proliferating, adipose cells undergo hypertrophy due to obesity, and there is evidence that adipose tissue becomes hypoxic due to increased tissue density (56). In obesity, adipose cells will upregulate genes associated with hypoxia, leading to activation of the hypoxia-inducible factor 1 (HIF-1) pathway. This creates an environment that drives persistent glycolysis and effector function in CD8^+^ T cells (55). This consistent stimulation may further drive the increase in exhausted phenotype seen in obesity models.

Furthermore, obesity leads to both systemic and tissue-specific inflammation. There are several mechanisms that contribute to this phenomenon, one of which is nutrient overload. Excessively high nutrient levels can cause oxidative stress through the release of reactive oxygen species and may also lead to endoplasmic reticulum (ER) stress. Increased oxidative and ER stress, combined with the activation of Toll-like receptors by fatty acids and glucose, will cause an inflammatory response which will further stimulate T cells (57).

Though the nutrient deprivation seen in cancer is not present in obesity (and in fact, nutrient levels are generally chronically elevated), persistent stimulation and hypoxia are evident. These two factors may therefore drive the presence of elevated levels of exhausted T cells in obese individuals, as demonstrated in murine models. Much of the existing literature on nutrient metabolism in cancer and obesity focuses on ATP-generating molecules, such as glucose, glutamine and fatty acids (58–62). Here, we consider an alternative nutrient that is often overlooked yet has a critical role in cellular proliferation, which is central to both cancer and the resulting immune response: cholesterol.

### 3.2 Cholesterol immunometabolism and anti-tumour response

Cholesterol metabolic reprogramming is also a hallmark of cancer (63). Cholesterol is required to facilitate cell division, and so to enable the rapid division of tumour cells, cancer cells employ tactics to circumvent regulation of cholesterol synthesis and uptake and maintain high levels of intracellular cholesterol. Cholesterol is different to other metabolites, as it cannot be catabolised and used for the production of ATP, and it is therefore not an energy source for cells. While critical for cell proliferation, cellular cholesterol accumulation is toxic, and so both the biosynthesis and uptake of cholesterol by cells is very tightly regulated.

**Figure 3:**
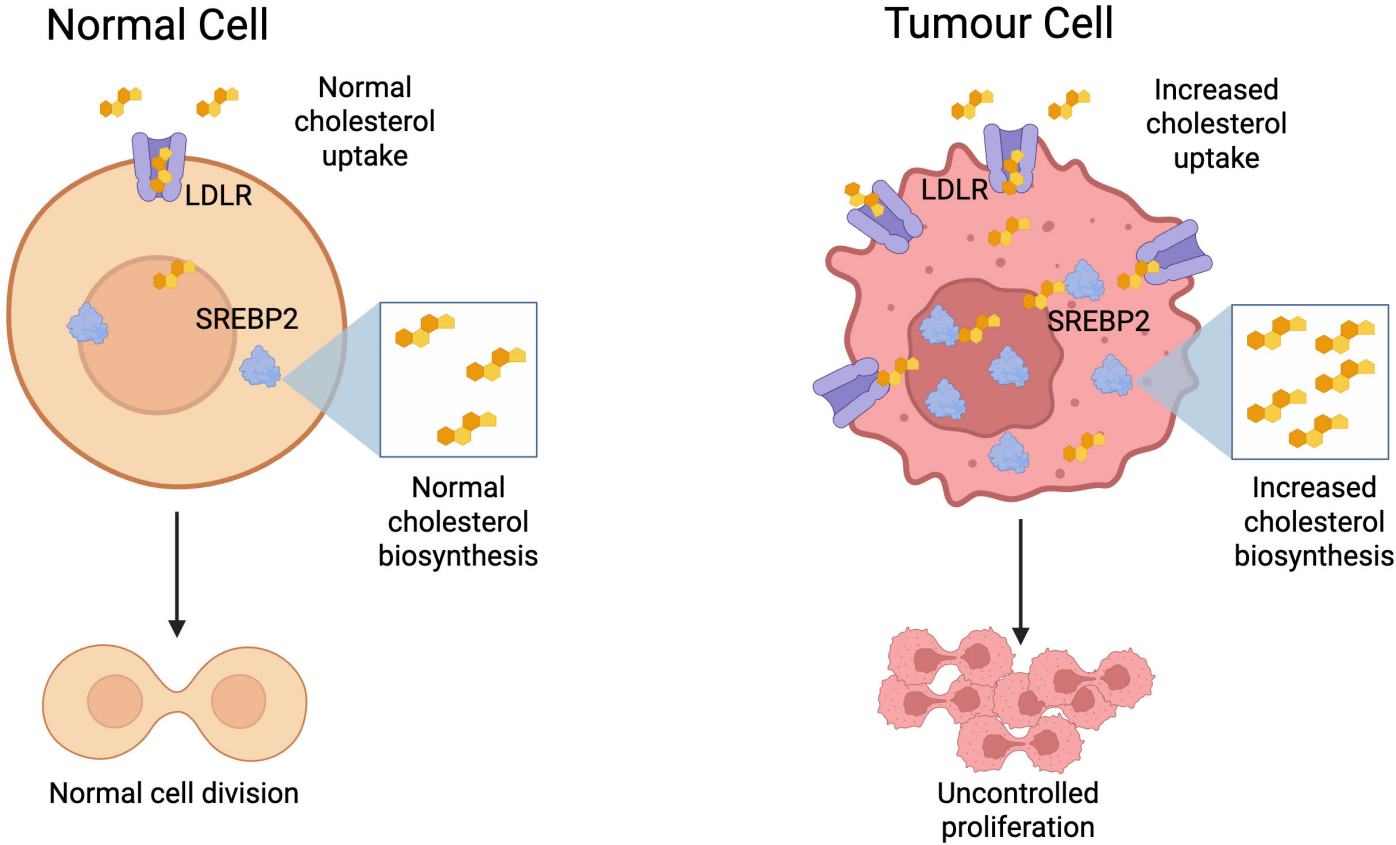
Metabolic reprogramming of tumour cells to facilitate increased cholesterol biosynthesis and uptake, enabling uncontrolled proliferation. Created with BioRender.

Cells in a wide range of cancers have been shown to overexpress the low-density lipoprotein receptor (LDLR) involved in cellular uptake of cholesterol, and sterol regulatory element-binding protein 2 (SREBP2), the master transcription factor that promotes cholesterol biosynthesis and import (64). Crucially, it was also shown that levels of these proteins did not decrease in the presence of exogenous cholesterol as they did in normal cells (65, 66, p. 19; 67). This enables cancer cells to circumvent the feedback loop that would normally inhibit cholesterol synthesis and uptake in the presence of high cholesterol levels. As a result, solid tumours are characterised by high cholesterol levels (68), and this dysregulation also leads to raised cholesterol levels in the TME (69) (**Figure 3**).

Together with SREBP-2, Liver-X-Receptors (LXR) are transcription factors critical to cholesterol homeostasis, activated by oxysterols (70). Dysregulation of LXR expression and activation is implicated in different cancers, though with variable mechanisms. For example, LXRs are known to be highly activated in triple-negative breast cancer tumour associated myeloid cells, which regulates macrophage activity and leads to an anti-inflammatory effect, reducing tumour killing (71). However, LXR activation may actually be beneficial in some cancers, with LXR activation leading to cell cycle arrest in prostate cell cancer lines (70). This may be due to the role of LXR in androgen production, which is closely linked with prostate cancer development. What is known is that during cell proliferation, crucial to both the immune response and cancer growth, the link between increased intracellular cholesterol and LXR activation is dysregulated, which means normal cholesterol efflux mechanisms are disrupted (72). While this pathway may therefore be a target to reduce proliferation of cancer, this may also interrupt the tumour immune response which heavily relies on proliferation of cancer-specific T cells.

As with all cells, cholesterol is essential to the healthy functioning and division of T cells. Upon activation, T cells will reprogram their metabolism to increase cholesterol synthesis and uptake, facilitating their rapid proliferation (73). However, as described above, excessive cholesterol accumulation is toxic and can lead to ER stress and cellular dysfunction (74). In the TME specifically, it has been found that elevated cholesterol leads to exhaustion of CD8^+^ T cells (69). Beyond the cancer context, hypercholesterolaemia has been shown to be linked to changes in T cells. In mice, hypercholesterolaemia has been associated with a T cell inflammatory response (75, 76). While it has not been shown that this progresses into T cell exhaustion, it is known that chronic stimulation contributes to the development of exhaustion. It is therefore possible that T cell activation induced by systemic hypercholesterolaemia could induce the same exhaustion phenotype seen in cancer.

Obesity is associated with an increased risk of hypercholesterolaemia (77), so this mechanism of T cell activation (and potentially exhaustion) may be present in a higher proportion of individuals with obesity compared to non-obese individuals.

Both obesity and cancer appear to lead to T cell exhaustion, through some similar mechanisms (e.g, hypoxia and chronic stimulation), and some differing pathways (e.g, nutrient deprivation vs. nutrient overload). Given that the function of exhausted T cells is rescued with checkpoint inhibitor treatment, it is possible that the paradoxical improvement in checkpoint inhibitor (CPI) outcomes in obese patients is influenced by an increase in the dysfunctional T cell compartment in obesity.

## 4 Immunotherapies and immunometabolism

### 4.1 Harnessing the immune system for cancer treatment

Though immunotherapy has experienced a renaissance in recent years, the concept of using the immune system against cancer originates in the late 19^th^ century. William B. Foley, a bone surgeon, found that when his sarcoma patients developed post-operative infections, their remaining tumours began to shrink. By injecting them with a cocktail of sepsis-inducing bacteria, Foley was able to induce anti-tumour immune responses and achieve durable remission in some of his patients. However, the risks associated with the treatment meant that immunotherapy was omitted from the arsenal of cancer treatment for almost a century in favour of radiotherapy and resection (78).

While some anticancer immunotherapies were developed in the 1980s (e.g, high-dose IL-2 treatment), a 1996 pre-clinical study published by Leach et al. (79) marked the start of the immunotherapy boom. This study concerned cytotoxic T-lymphocyte-associated protein 4 (CTLA-4), an immune checkpoint expressed on T cells which binds to ligands CD80 and CD86. These ligands normally bind to CD28, leading to co-stimulation which, alongside antigen presentation to the T cell receptor (TCR), facilitates T cell activation. CD28:CD80/86 binding also leads to T cell proliferation, increased longevity and differentiation. However, these ligands preferentially bind to CTLA-4, so when it is presented, these functions are inhibited. Overall, this leads to a reduction on the effector response of T cells. CD80 and CD86 are generally presented by antigen-presenting cells in secondary lymphoid organs (lymph nodes and spleen), and so CTLA-4 is thought to regulate the T cell response early in the immune process (80). The Leach study found that CTLA-4, which had been identified just a year prior, could be exploited to improve the anti-tumour immune response in mice. Leach found that by injecting mice with anti-CTLA-4 antibodies, tumour regression improved compared to untreated or anti-CD28 treated mice in a range of tumour types (79).

In 2002, the Honjo lab at Kyoto University discovered that another immune checkpoint, PD-1, could be a beneficial target for cancer immunotherapy along with its ligand, PD-L1 (81). PD-1 is present on the surface of a range of immune cells, including T cells (especially post-activation). When presented on T cells and bound to its ligand (PD-L1 or PD-L2), PD-1 causes downstream signalling that leads to both direct and indirect inhibition of TCR signal transduction, reducing activation of T cells. Additionally, PD-1 binding may reprogram T cells from glycolysis-led metabolism, indicative of an effector phenotype, to greater reliance on fatty acid oxidation, which is representative of memory phenotype (82). Together, these effects lead to reduced T cell effector function. This pathway is crucial to maintaining peripheral tolerance, thought there is increasing evidence for PD-1’s importance in the lymph node (83). The Honjo lab demonstrated that cytotoxic T cells were less effective at tumour killing & lysis when presented with a PD-L1 presenting tumour model *in vitro*. Furthermore, the same paper demonstrated that the growth of myeloma tumours in mice was inhibited with administration of PD-L1 blockade, and completely halted in PD-1 deficient mice. Later mouse studies showed that PD-1 was highly expressed on dysfunctional T cells following chronic infection (84, 85) and that PD-1 or PD-L1 blockade improved the function of these T cells (84). Together, this work showed promising evidence that PD-1/PD-L1 blockade could effectively inhibit tumour growth and help recover the function of chronically stimulated T cells.

In the clinic today, anti-PD-1/PD-L1 and anti-CTLA4 are commonly used checkpoint inhibitors for a range of cancers, though recently the first LAG-3 inhibitor has also been approved for clinical use (86). These drugs work by binding with the immune checkpoints on T cells without inducing the downstream signalling that would normally lead to suppression of effector function and killing ability. Studies have shown that these drugs are able to recover function in T cells that had previously been exhausted due to chronic stimulation and exposure to the tumour microenvironment (TME) (87, 88).

Surprisingly, there is currently little information regarding the effect of checkpoint inhibitors on systemic metabolism. A 2021 study of 374 cancer patients found that overweight patients with high metabolic risk were more prone to higher severity toxicities when taking immune checkpoint inhibitors, and that this additional risk was not present with either obesity or reduced metabolic risk (89). It is clear therefore that changes in systemic metabolism have an impact on the function of CPIs and other T cell therapies. However, the inverse, that is, the impact of CPIs on systemic metabolism, has not been explored, and would be an interesting topic for further investigation.

### 4.2 Human data: obesity and lipid paradoxes

CPIs have been transformational in oncology treatment, and 2020 estimates suggest that 36-39% of all patients with cancer in the US are eligible for CPI treatment (90). Hundreds of thousands of patients worldwide have been treated with these drugs since the approval of anti-CTLA-4 agent Ipilimumab in 2011. With this large patient cohort, it is now possible to understand how the effects of these drugs change depending on patient characteristics.

In 2018, a particularly surprising story began to emerge. McQuade et al. published unexpected findings from a cohort of 1918 patients that obese male melanoma patients had improved progression-free survival after treatment with checkpoint inhibitors (91). Over the following years, this pattern was also seen in melanoma patients regardless of sex (92), and in non-small cell lung cancer (NSCLC) (93). Obesity is defined here as body mass index (BMI) over 30. Given that obesity generally increases the risk of cancer development, this was considered an unexpected finding and named the ‘obesity paradox’. Similar inverse relationships have also been reported in cancer beyond the CPI context, where patients undergoing chemotherapy who responded to treatment showed increases in serum total cholesterol and LDL levels from baseline and above healthy levels (94).

Several hypotheses have been suggested to explain the obesity paradox. The effect may be related to increased T cell exhaustion which is often seen in cases of chronic antigen presentation, such as in cancer, obesity and autoimmunity, or is possibly related to improved immune infiltration in obesity (95). As well as exhaustion, an increase in tissue-resident memory T cells (T_RM_s) may also be implicated in the obesity paradox. T_RM_s are a T cell subset which resides in tissues rather than circulating through the body. These cells are characterised by their high expression of immune checkpoints, and increased effector function (96). For these reasons, there are several studies that suggest a link between CPI treatment efficacy and heightened levels of T_RM_s (97, 98). Interestingly, T_RM_s also appear to be upregulated in the adipose tissue of individuals with obesity (99). It is therefore possible that the obesity paradox may be partly explained by an increase in the T_RM_ compartment in obese individuals. Despite these hypotheses, as yet there is no confirmed answer explaining the obesity paradox effect. However, CPI treatment in obesity is not the only context in which we see an interplay of metabolic dysregulation and immune response.

Interestingly, paradoxical associations between cholesterol levels and immune activation have also been reported in the context of chronic inflammatory diseases. The lipid paradox refers to an unexpected observation made nearly 15 years ago describing the paradoxical relationship between systemic lipid levels and disease activity in patients with Rheumatoid Arthritis (RA), a common chronic inflammatory autoimmune disorder (100). Cardiovascular disease is commonly associated with elevated lipid levels in serum. However, in RA decreased systemic levels of total cholesterol and LDL are associated with its hyperinflammatory state (100), and lipid concentration is inversely associated with inflammatory markers (101). Even more interesting is the fact that upon anti-inflammatory treatment, lipid levels increase (or normalise) and cardiovascular and metabolic health improves (100, 102, 103). This paradoxical relationship is thought to be due to the chronic inflammatory nature of RA, pointing out at the fact that chronic exposure to pro-inflammatory cytokines dissasociates plaque formation from hyperlipidaemia.

With the clear link between metabolic dysregulation (both in the case of obesity and dyslipidaemia) and the immune response, it is very likely that systemic metabolism is influential in the mechanism and efficacy of immune-modulating drugs that are a burgeoning part of both cancer and autoimmunity treatment. While overweight and obese patients respond better to CPIs, they also have a greater susceptibility to cancer, with obese people more likely to develop 13 types of cancer than those with a healthy BMI (104). It should be noted that BMI (which is generally used to classify obesity) is a non-specific measurement which does not take into account body composition (e.g, fat vs muscle mass). These additional factors can also have a significant effect on cancer treatment outcomes.

### 4.3 Body composition and nutritional status.

**Figure 4:**
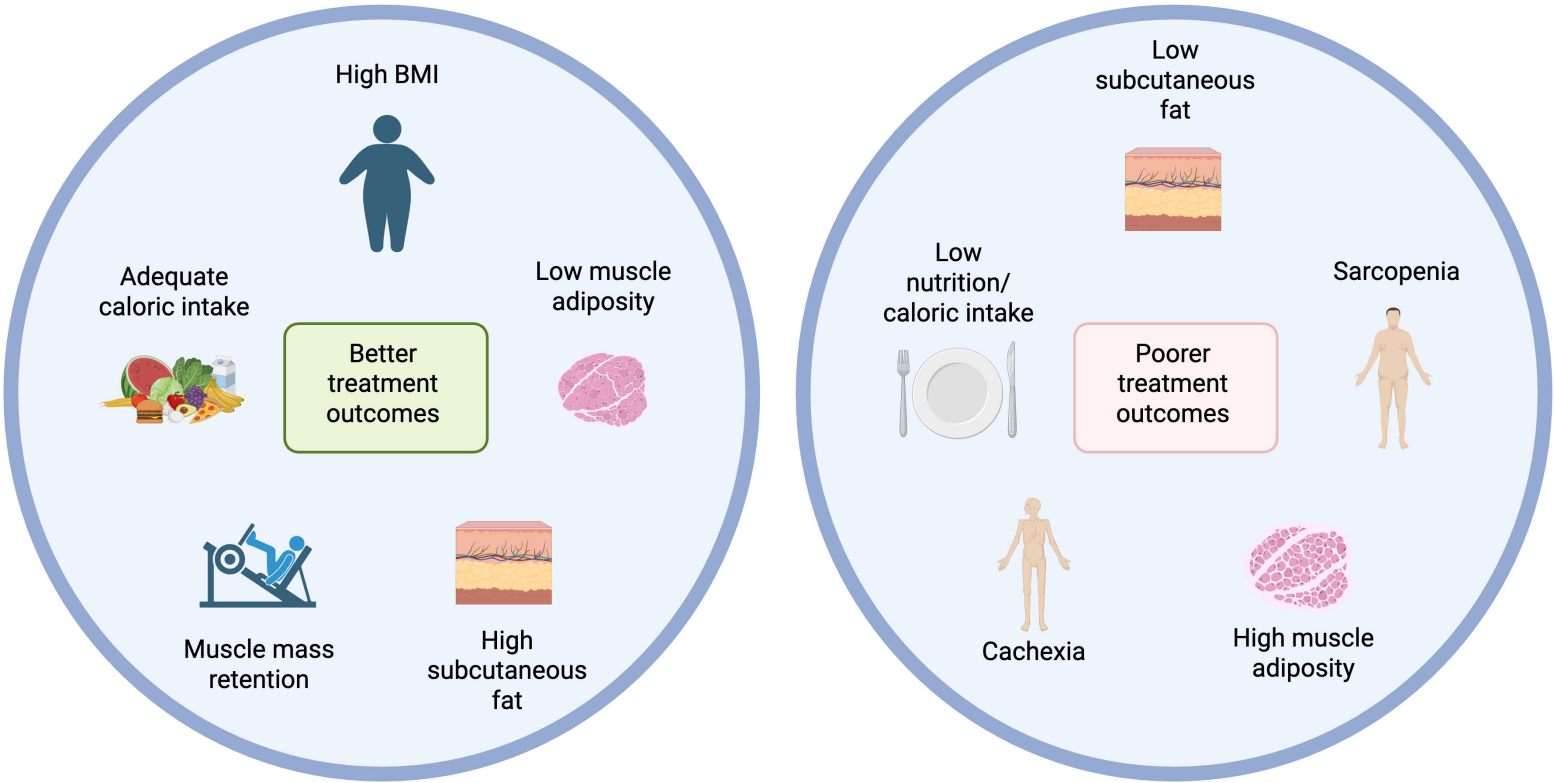
Body composition and nutritional influences on immunotherapy outcomes. Created with BioRender.

Although the “obesity paradox” in cancer immunotherapy is widely discussed, a major limitation of many supporting studies is their reliance on BMI, rather than direct measures of body composition. BMI fails to distinguish between fat types or lean mass and is thus confounded. In contrast, emerging evidence suggests that skeletal muscle and subcutaneous fat are critical predictors of immunotherapy response. **Figure 4** gives an overview of the main factors relating to body composition and nutrition currently known to contribute to immunotherapy outcomes.

Skeletal muscle mass has consistently shown prognostic significance across multiple cancer types. In gastric cancer, low skeletal muscle index (SMI) or sarcopenia is associated with worse survival (105, 106) and reduced tumour regression in patients receiving immunotherapy alone or with chemotherapy (107). Similar associations are seen in non-small cell lung cancer (NSCLC) (108–110), small cell lung cancer (SCLC) (111), hepatocellular carcinoma (HCC) (112, 113), melanoma (108, 114), head and neck squamous cell carcinoma (HNSCC) (115), where low SMI, muscle density, or sarcopenia predict poorer progression-free survival (PFS) and overall survival (OS) following CPIs. In renal cell carcinoma (RCC), SMI was the only body composition metric linked to OS after CPI therapy, with transcriptomic data showing that tumours from low-SMI patients had increased angiogenic and inflammatory signatures (116). These findings seem to be supported by other studies where poor body composition risk score (117) or higher cachexia index (118) had worse OS and PFS. Together, these studies demonstrate that low muscle mass and sarcopenia are prognostic factors affecting immunotherapy efficacy and long-term survival of cancer patients. Retaining muscle mass during treatment is then imperative, as loss of skeletal muscle during systemic therapy, especially in male NSCLC patients, further predicts poor prognosis (119).

Nutritional and metabolic status also influence outcomes. The ELY-2 study (120) in metastatic NSCLC patients under anti-PD1 therapy found that hypermetabolic patients who met ≥90% of their caloric needs had longer PFS, highlighting that adequate caloric intake may mitigate the negative impact of elevated resting energy expenditure. Nutritional indices like the Geriatric Nutritional Risk Index (GNRI) and Prognostic Nutritional Index (PNI) are also prognostically valuable. GNRI was associated with longer OS and PFS, particularly in older and early-stage NSCLC patients (121), while obesity was linked to poorer outcomes. Similarly, low PNI and low total adipose tissue predicted worse survival in NSCLC patients receiving CPIs and radiotherapy (122). Furthermore, Ding et al. found that malnutrition, sarcopenia, and low advanced-lung-cancer-inflammation-index scores were independently associated with poor survival in response to chemo-immunotherapy (123). Altogether, integrating GNRI, PNI, and body composition analysis enhances prognostic accuracy and supports more personalized treatment strategies.

Targeting amino acid metabolism through dietary manipulation or supplementation may be a strategy to simultaneously enhance immune-mediated tumour control and address cancer-associated metabolic dysfunctions. In a preclinical study, β-hydroxy-β-methylbutyrate (HMB), a leucine metabolite with muscle-sparing and immunomodulatory effects, reduced tumour growth, preserved muscle mass in obese mice, and improved anti-PD1 response in lean mice (124). These results underline HMB’s potential to not only support immune-based therapies but also ameliorate cancer cachexia, which makes it an attractive candidate for integrated cancer care.

Subcutaneous fat is another favourable prognostic factor. In gastric cancer, low subcutaneous fat area (SFA) or index (SFI) predicts poorer OS and treatment response in patients treated with CPIs (105, 106, 125) combined with PNI and SMI, high SFA further stratifies patients with better outcomes (105). However, fat and muscle loss during treatment still correlates with progression risk. Loss of subcutaneous adipose tissue and sarcopenia after beginning CPI treatment is correlated with higher risk of disease progression in metastatic cancer patients (126). In NSCLC, low SFI predicts shorter OS and PFS (109), while in RCC, high subcutaneous fat percentage (SAT%) correlates with improved PFS and increased intratumoral PD1^+^CD8^+^ T cell density (127), potentially explaining the better therapeutic outcome following CPI treatment. Yet, some studies found no significant associations between adiposity and outcomes in RCC (116) and melanoma (114). Overall, subcutaneous fat appears to be a reliable and favorable prognostic indicator in CPI-treated patients, although this association is influenced by cancer type and individual patient characteristics.

On the other end, myosteatosis and intramuscular adipose content (IMAC) tend to be associated with worse outcomes. In urothelial carcinoma (128) and NSCLC (110, 129), high levels of myosteatosis correlates with lower OS and PFS, while better muscle quality (low IMAC) is associated with improved outcomes.

Compared to subcutaneous fat and muscle, visceral fat shows inconsistent correlations. While low visceral adiposity has been linked to poor disease control in some cancers (HNSCC, HCC, colorectal, and urothelial) (112, 115, 128, 130), findings remain mixed. In one NSCLC study, higher visceral fat index (VFI) predicted worse survival (93), but results weren’t consistently replicated.

The emerging evidence strongly supports the notion that body composition, particularly skeletal muscle mass and subcutaneous fat, plays a critical role in determining outcomes for patients undergoing cancer immunotherapy. While BMI has traditionally been used as a surrogate for obesity, it remains a poor proxy for true body composition, failing to differentiate between adiposity and lean mass. Importantly, a high BMI may reflect not only increased fat mass but also greater muscle mass, both of which can confer prognostic value. This complexity challenges the oversimplified narrative of the obesity paradox. As such, reliance on BMI alone obscures the nuanced interplay between fat distribution, muscle integrity, and treatment response. Moreover, attention has to be paid in maintaining nutritional status during treatment, and routine nutritional assessment should be a key component of immunotherapy management. Moving forward, integrating direct body composition assessment with nutritional markers will be essential for optimizing patient stratification, guiding supportive care interventions. In the following sections, we will consider potential adjuvant interventions that modulate systemic metabolism, with the ultimate goal of improving efficacy of immunotherapies.

### 4.4 Animal models vs human immunometabolism

Much of the existing literature exploring the immunometabolic effects of checkpoint inhibitors relies on animal models, predominantly using rodents. While animal models provide a unique opportunity to study the systemic effects drugs *in vivo*, there are significant differences between human and rodent systems that should be considered.

#### 4.4.1 Immune system differences

While human and rodent immune systems contain most of the same cell subtypes, the frequencies of these vary widely. **Table 1** shows a comparison between immune cell subtypes in whole blood in humans (132) and in young mice (131). It should be noted that there will be variances in the exact composition of immune cell subtypes between individuals based on a range of factors including age and gender, and that these can be even more pronounced in mice due to the availability of different strains (134). However, the overall differences in immune composition between humans and mice remain similar.

**Table 1:** Percentage of various immune cell subtypes as a percentage of leukocytes in peripheral blood of 3-month-old male and female C57BL/6J mice (131) and healthy human donors between 21–48 years old (132).

Cell type	% Frequency – mouse (median, range)	% Frequency – human (median, range)
Lymphocytes	74.285 (62.4-81.8)	44.3 (40.7-59.5)
T-cells	23.23 (18.2-30)	60.6 (48.7-77.1)
CD4^+^ T-cells	53.185 (48-58.4)	35.46 (26.15-48.26)
CD8^+^ T-cells	7.44 (31-44.5)	21.13 (12.38-33.11)
B-cells	46.06 (36.9-52.5)	13.15 (6.36-16.51)
NK cells	4.765 (3.6-7.5)	15.41 (6.84-35.17)
NKT cells	2.7 (0.6-6.3)	2.35 (0.26-4.42)
Monocytes	9.61 (7-13.8)	9.28 (6.04-11.56)
Dendritic cells	0.67 (0.3-1.15)	1.16 (0.63-1.46)
Eosinophils	3.065 (1.7-4.08)	0-4*
Neutrophils	9.17 (4.9-21.7)	40-60*
Basophils	0.705 (0.38-1.01)	0.5-1*

Values are given as a median percentage, with the range percentages given in brackets. Values marked with an asterisk (*) (human eosinophil, neutrophil and basophil percentage ranges) are laboratory reference ranges from (133). T cells, B cells, NK cells and NKT cells are given as a percentage of the lymphocyte compartment. CD4^+^ and CD8^+^ T-cell values are given as a percentage of the T-cell compartment. For mouse values, n = 12 individual mice, for humans n = 9 individual donors.

One of the most notable differences is the lymphocyte vs neutrophil frequency. In mice, nearly three-quarters of the total leukocyte compartment is comprised of lymphocytes, with B-cells being nearly twice as common as T cells. In humans, closer to half of leukocytes are lymphocytes, with approximately half being neutrophils. Neutrophils are much less prominent in mice, making up only around 10% of leukocytes. While the impact of the differences in neutrophils vs lymphocytes in mice is still being discussed (135, 136), as CPIs are known to target T cells, the relative enrichment in this compartment in rodents must be considered as a potential limitation of murine models in this context.

Considering the T cell compartment specifically, differences exist in the proportion of memory T cells. Humans have far higher levels of CD8^+^ effector memory cells in blood than laboratory mice, and more antigen-experienced T cells in general (137). This is possibly due to the clean environment that laboratory animals are housed in, as this study showed that non-laboratory mice had an increase in antigen-experienced T cells compared to their laboratory counterparts. However, as experimental animals must be housed in a clean environment, the issue of reduced effector and memory T cell populations remains.

Furthermore, this same study demonstrated that mice had almost no CD8^+^ T cells in the cervix, where these cells were abundant in humans. This evidences that there is less infiltration of cytotoxic T cells in mice in at least one tissue, and this could have a critical impact on the mechanism of checkpoint inhibitors and their tissue-specific effects.

#### 4.4.2 Metabolism differences

As well as the immune system, rodents and humans have critical differences in their metabolism. Considering metabolism holistically, the basal metabolic rate (BMR) of rodents and humans varies dramatically, with mice and rats having a BMR per gram of body weight around 7 and 6.4 times higher than humans respectively (138, 139).

In addition, mice also respond far more dramatically to calorie restriction than humans due to increased cellular metabolic instability. In mice, caloric restriction (which is known to increase cellular metabolic stability (140) increases lifespan significantly (35-65%) (141). In non-obese humans however, where cells are more metabolically stable, caloric restriction has only a moderate effect (3–5 years, or around 5.5%) (139). The inverse is seen when considering high-fat diets in mice. Mice fed a high-fat diet (HFD) had a median reduction in lifespan of 77 days, equating to around an 11% reduction in lifespan (142). In humans, moderate obesity is associated with around 2–4 years reduction in lifespan (or approximately 4.1%). It should be noted that severe obesity (BMI < 40 kg/m2) is associated with a similar lifespan reduction in humans as seen in HFD mice, but this is still uncommon in the general population (143). The differential effects of both calorie restriction and nutrient overload in mice and humans are of particular relevance to murine obesity models, and should be carefully considered.

Considering cholesterol, mice and rats also have a very different cholesterol profile than humans. While humans tend to have most of their cholesterol in plasma as low-density lipoprotein (LDL), rats and especially mice have a higher proportion of high-density lipoprotein (HDL) (144). The effects of cholesterol-modulating interventions in rodent models may differ from the effects in humans due to this varying lipid profile. Any changes to cholesterol levels in mice resulting from experimental interventions should be analysed with this in mind, particularly as a non-optimal total cholesterol/HDL ratio is associated with increased mortality in humans (145).

There are several other critical differences that researchers should consider when using murine models for immunometabolism studies. For example, vitamin C is a widely-researched antioxidant that is known to be involved in immune system regulation (146). While humans must get their vitamin C from dietary sources, mice can make vitamin C endogenously (147). Mouse studies exploring vitamin C supplementation should therefore explore whether supplementation impacts endogenous production, to understand the impact of this potential confounder. Finally, of particular interest for this review is the differences in the gut microbiome of humans and mice. While the murine digestive system is similar in many ways to that of humans, one notable difference is the relatively larger cecum in mice. The cecum is a key site for fermentation of plants and is also important for vitamin B and K production (148). This increased fermentation of ingested food in mice enables them to access different nutrients via digestion than humans and is likely involved in supporting the different bacterial strains seen in the gut microbiota of mice compared to humans. Nutrition and the gut microbiome is increasingly reported to be involved in systemic immunity (149, 150), and so these differences cannot be overlooked.

While *in vivo* models provide an invaluable opportunity to understand the mechanisms of immunometabolic interventions at a systemic level, there remain critical differences between murine models and humans. Researchers must therefore look for ways to optimise their models to mitigate the impact of these differences and create a more representative *in vivo* system.

#### 4.4.3 Optimising murine models

To run obesity studies, mice were historically generally fed a HFD *ad libitum*, leading to weight gain. However, mouse models deficient in leptin have now been developed. Leptin is a hormone involved in regulation of appetite, and leptin-deficient mice will therefore consume increased food compared to wild-type mice, leading to obesity (151). This is often considered to be a more representative model, as while obese humans are not generally leptin-deficient, they are leptin-resistant, meaning that higher levels of leptin are required to produce the same appetite-suppressive effects as in lean people (152). Furthermore, the gut microbiota of leptin-deficient mice potentially have more similarities to obese humans than wild-type counterparts, with the gut of leptin-deficient obese mice producing more monosaccharides and short chain fatty acids compared to lean controls (153), which is also seen in humans (154). However, leptin-deficient mice show pathology that is not seen in humans, particularly profound insulin resistance and growth limitation (155). This distinct pathology limits the model’s translatability to humans.

Considering the immune system as a whole, the limitations of murine models have long been understood. Efforts to overcome this have led to the development of mice with humanised immune systems. Initially, this involved taking immunodeficient mice and introducing human immune cells. However, there are several issues with this model, including reduced lifespan and insufficient immune responses to pathogens (156). More recently, the THX mouse strain has been developed that has a full and functioning human immune system, accompanied by a human-like gut microbiome (157). While this represents a significant advancement in murine models, the conditioning process to develop these mice requires administration of 17β-estradiol (E2). E2 is a sex hormone known to impact the expression and production of cytokines in epithelial cells during the response to infection (158), and plays a key role in the reproductive cycle. With the THX mouse model being relatively new, it is not yet known how, or if, the use of E2 in model development will confound immunological findings.

While improved murine models are breakthroughs in providing more representative *in vivo* models of human immunometabolism, there remains fundamental differences in murine and human biology. While murine models are still critical to immunometabolic research, scientists and clinicians can also consider ways that they might optimise metabolic interventions in the human and better leverage clinical data.

#### 4.4.4 Optimising clinical data and interventions

Since the introduction of Ipilimumab in 2011, hundreds of thousands of patients have been treated with checkpoint inhibitors. Due to this large, diverse patient cohort, there is an opportunity to assess the impact of metabolic interventions, in many cases without requiring the patient to make any changes to their normal routine.

Of relevance to this review are gleaning greater insights into the effects of personalised nutrition interventions on CPI response. Though there remains some access challenges in low-income countries (159), CPIs are now used globally to treat a range of different cancers. This global reach means that patients will naturally have a range of different diets (e.g, standard western diet, Mediterranean diet, vegan or vegetarian etc.) By gathering information on diet from patients undergoing CPI treatment, it may be possible to undertake larger-scale analysis to understand whether responses are correlated with a particular diet. If patients are consented to provide samples (such as blood, faeces or urine), metabolomic analysis could be used to delve further into the mechanisms behind any identified benefits, as has been done in other disease contexts such as cardiovascular disease (160) and rheumatic disease (161).

The impact of cholesterol-modulating drugs on CPI response and the mechanisms underlying this have also yet to be fully defined. Dyslipidaemia is very common in the western world, affecting approximately 11.3% of adults in the US (162). This means that within the CPI patient cohort, many will have high cholesterol, many will be on statins or other cholesterol-modulating drugs, and others will have normal cholesterol levels. While there is already data exploring the correlation between cholesterol, statins and CPI response, the mechanism behind this remains unclear. A combination of metabolomics and RNA-sequencing of CPI patients could provide greater insights into the interplay between cholesterol metabolism and CPI response, and potentially assess whether statins alone are beneficial without associated high-cholesterol. Additionally, this analysis could potentially elucidate predictive biomarkers to identify those who are likely (or not) to respond to treatment.

The large, global and expanding cohort of CPI patients provides a wealth of data that should be leveraged further to understand the impact of diet and nutrition, as well as other metabolism-modulating interventions. With further research, it may be possible to identify predictive biomarkers that enable personalised dietary and medicinal interventions to improve individual responses to treatment. The following section considers metabolic interventions that have already been identified as affecting the immune system, and may therefore impact the efficacy of CPI treatment.

## 5 Adjuvant interventions – targeting metabolism to boost the anti-tumour immune response

Immunotherapies are now a cornerstone of cancer treatment, and as previously mentioned, over a third of cancer patients in the US are eligible for these drugs (90). However, despite widespread eligibility, only around 10.9% of patients respond positively to CPI therapy (90). This varies significantly between cancer type, but the fact remains that in most cases, only a minority of patients will benefit. It is therefore critical to consider adjuvant interventions which may improve the efficacy of CPIs, and therefore outcomes for patients. With a clear link between systemic metabolism and the anti-tumour immune response, here we consider metabolic interventions that could potentially be used alongside CPI therapy to improve treatment response.

### 5.1 Statins

Statins are a class of drugs used to lower serum cholesterol levels. They work by inhibiting HMG-CoA reductase, an essential element of the mevalonate pathway which is upstream of cholesterol biosynthesis. As a result, people taking statins will produce less endogenous cholesterol (163). Statins are generally prescribed to people with hypercholesterolaemia to reduce their cholesterol levels.

Figures from 2008 state that approximately 39% of the global adult population have elevated cholesterol levels (164) and more recent estimates put this figure at around 53% in the UK (165). The prevalence of the condition gives us an opportunity to compare the outcomes of patients on CPI treatment with and without high cholesterol and associated medications. Analysis has found that hypercholesterolaemia is associated with improved CPI outcomes (such as overall survival and reduced all-cause mortality) (166–168), with a potentially increased benefit in those receiving PD-1 blockade (169). It has been suggested that this effect may be due to the proinflammatory effects of increased cholesterol supporting the mechanism of CPIs (167).

Interestingly, though statins reduce cholesterol levels, their use is also correlated with improved CPI outcomes in humans (170, 171). Similar results are seen with ezetimibe (another cholesterol lowering medication) (166). At present, statins are only indicated for lowering cholesterol levels in those with elevated cholesterol, so it is not clear whether statins themselves improve CPI outcomes, or whether elevated cholesterol levels lead to long-term effects on CPI efficacy that persist despite treatment.

There is some evidence that statins modulate the immune response. It has been shown that inhibiting cholesterol biosynthesis with either atorvastatin or 25-hydroxycholesterol prevents CD4^+^ T cells from switching from a pro-inflammatory to anti-inflammatory phenotype (172). As a result, statins may slow the resolution of the immune response, which is key in the cancer context where there is chronic tumour-associated antigen presentation. Additionally, statins are known to have an antiproliferative effect, which aligns with the necessity of cholesterol for cell division (173). This could be a double-edged sword for CPI treatment, as this may affect proliferation of both cancer cells and cytotoxic T cells. The impact of this mechanism on CPI efficacy is therefore unclear.

What is clear is that statins do not negate the positive effect on CPI efficacy conferred by hypercholesterolaemia. To understand whether cholesterol-lowering drugs alone benefit CPI efficacy, it would be necessary to assess their effects in humans outside the context of hypercholesterolaemia.

### 5.2 GLP-1 receptor agonists

Glucagon-like peptide 1 (GLP-1) is an endogenous hormone that binds to the GLP-1 receptor. The GLP-1 receptor is expressed on a wide range of human cells, and the impact of receptor binding is tissue-specific. Notably, binding of GLP-1 to pancreatic β-cells facilitates insulin release. In the gut, GLP-1 binding inhibits gastric emptying and gut motility, which reduces appetite and slows the rate of glucose absorption after eating. These effects in particular have led to the development of GLP-1 receptor agonists (GLP-1RAs) which are used for the treatment of both obesity and diabetes (174).

*In vitro*, GLP-1RA treatment is associated with a reduction in the inflammatory response, particularly macrophage and monocyte mediated inflammation (175). Considering the T cell compartment, exhausted CD8^+^ T cells have been shown to express GLP-1R, and there is evidence that it behaves as a negative co-stimulatory molecule, reducing T cell effector function when bound to its ligand (176). These results present GLP-1RAs as anti-inflammatory treatments which affect a range of immune cells, and this may indicate a potential negative impact on the efficacy of CPIs.

There is a relative wealth of *in vivo* mouse studies investigating the impact of GLP-1RAs in the general cancer context, though it is conflicting. Growth of prostate cancer was reduced in both *in vitro* models using prostate cancer cell lines, and in *in vivo* mouse models with administration of exendin-4, a GLP-1RA (177). GLP1-RAs plus metformin treatment have also been shown to have antitumour effects in pancreatic cancer *in vitro* (178) and breast cancer both *in vitro* and with *in vivo* mouse models (179). Contrastingly, GLP-1 receptor agonism has been shown to increase proliferation of neuroendocrine tumour cells both *in vitro* and in mouse xenograft models (180). Furthermore, liraglutide (a GLP1-RA) has been shown to cause thyroid C-cell proliferation in rats, the cell type from which medullary thyroid cancer originates – however, this effect was not seen in non-human primates (181). GLP-1 receptor expression varies extensively between tumours, and it’s use as a prognostic marker also depends on cancer type (174). This may explain the differences in the antitumour (or pro-tumour) effects of GLP-1RAs in different cancer models.

There is limited data available concerning the impact of GLP-1RAs on the efficacy of anticancer immunotherapies specifically. One mouse study found that liraglutide enhanced anti-tumour efficacy of PD-1 blockade via reduction of neutrophil extracellular traps, which are known tumour growth promoters (182). In humans, there is some evidence that cardiovascular IRAEs resulting from CPI treatment are reduced in those taking GLP-1RAs (183, p. 202), however, there is currently no evidence in humans regarding the efficacy of CPIs administered alongside GLP-1RAs.

It is clear that GLP-1RAs have systemic anti-inflammatory effects, and these may have an impact on CPI treatment by reducing cardiovascular IRAEs. However, it is currently unclear whether this would also attenuate the efficacy of CPIs in humans by inhibiting their proinflammatory mechanisms of action. Furthermore, GLP-1RAs appear to have varying impacts in different cancers. It remains to be seen whether this is also the case with differing CPI treatment regimes (e.g, PD-1 vs CTLA-4 blockade), or indeed whether the tissue-specificity of GLP-1 receptor binding influences the severity or location of IRAE development. With a growing cohort of patients taking GLP-1RAs for both diabetes and obesity, this presents an opportunity to assess their impact on CPI treatment and clarify the open questions in this area of research.

### 5.3 Nutritional strategies

Beyond supportive care, specific dietary and micronutrient interventions are emerging as modulators of the tumour microenvironment and immune response. Strategies like ketogenic and fasting-mimicking diets, along with vitamin and bioactive compound supplementation, show promise in enhancing CPI efficacy, reducing toxicity, and overcoming resistance. This highlights the growing relevance of nutrition in personalized immuno-oncology.

The ketogenic diet (KD) and fasting-mimicking diets (FMD) have garnered growing interest as adjunctive interventions in cancer treatment due to their potential to modulate tumour metabolism and enhance anti-cancer immune responses. Preclinical studies demonstrate that KD and its metabolite, 3-hydroxybutyrate (3HB), enhance anti-PD-1 and anti-CTLA-4 therapies and these effects depended on T cell-mediated immunosurveillance and involved modulation of the tumour microenvironment (184). In prostate cancer models resistant to immunotherapy, cyclic KD and ketone body supplementation epigenetically reprogram both tumour and immune cells (185). In RCC, KD and ketone bodies improved mitochondrial metabolism and sensitized tumours to anti-PD-L1 therapy (186). FMD also enhanced immunotherapy efficacy and reduced immune-related adverse events in triple-negative breast cancer models (187), supporting the dual benefit of boosting response while minimizing toxicity.

Vitamins further modulate immune responses. Vitamin D supplementation corrected hypovitaminosis in cancer patients and was associated with improved OS, prolonged time to treatment failure, higher disease control rates and of reduced thyroid-related immune adverse events (188). Furthermore, vitamin C synergizes with anti-PD1 therapy by enhancing CD8^+^ T cell and macrophage function and shifting macrophage polarization from immunosuppressive M2 to pro-inflammatory M1, which is crucial for sustaining effective antitumor T cell responses (189, 190). Folate metabolism, essential for immune cell function, is another key target. Pemetrexed, a folate inhibitor, promotes immunogenic cell death and enhances T cell mitochondrial activity when combined with CPI (191), and elevated B12 has been associated with worse outcomes in CPI-treated patients, suggesting a need for targeted modulation (192). Of note, both folate and B12 are essential cofactors in DNA synthesis, methylation, and cellular energy metabolism, which are critical to the proliferation and function of immune cells. However, disruption of folate pathways may enhance anti-tumour immunity by impairing tumour cell proliferation, while anti-folates can simultaneously support T cell activation by increasing the availability of 3-phosphoglycerolphosphate and other key metabolic intermediates, thereby improving T cell metabolic fitness as suggested by Schaer et al. (191). Future studies should aim to dissect the mechanistic underpinnings of these associations and explore how targeted modulation of B12 and folate metabolism might enhance treatment outcomes.

Finally, phenolic diterpenes, such as carnosic acid and carnosol from rosemary extract (SFRE), show synergistic effects with pembrolizumab and chemotherapy in NSCLC. Clinical data further demonstrated that SFRE can reduce inflammatory and metabolic markers linked to immune suppression such as MAPK, NLRP3 inflammasome, and SREBF1 suggesting that phenolic diterpenes can remodel both tumour metabolism and systemic immune pathways, potentially amplifying the anti-tumour immune response and improving patient outcomes (193).

Together, these findings underscore the multifaceted and increasingly recognised role of ketogenic and fasting-mimicking diets, as well as vitamins and phenolic compounds, as modulators of tumour metabolism, immune microenvironment and response to CPIs. As the field advances, integrating nutritional and metabolic profiling into immuno-oncology could pave the way for novel adjunctive strategies aimed at optimizing patient outcomes through precision nutrition.

## 6 Concluding remarks

Throughout this review, we have discussed the link between the dysregulation of systemic metabolism seen in obesity, and its clear impact on cancer development and treatment response. This is particularly highlighted by the obesity paradox in checkpoint inhibitor therapy, which sees obese individuals having better responses to treatment despite their greater propensity to develop cancer initially. We have explored potential mechanisms behind this paradox, including the increase in T cell exhaustion seen in obesity, as well as a potential for immune cell residency to play a key role. While high BMI is correlated with improved response to CPIs, there is a wealth of evidence that specific body composition measures, including muscle adiposity and subcutaneous fat, are better prognostic indicators of treatment outcome. These measures should therefore be considered alongside BMI.

While dysregulation in glucose levels is frequently discussed in the context of obesity, altered metabolism in obesity goes beyond energy production. Cholesterol is a critical metabolite which is involved in both the development of cancer and the immune response, and is frequently dysregulated in obesity. Both immune cells and cancer cells will produce and uptake increased levels of cholesterol to support their proliferation, but where immune cells are prone to endoplasmic reticulum stress due to this reprogramming, this does not affect cancer cells in the same way. Furthermore, hypercholesterolaemia seems to be correlated with improved outcomes in checkpoint inhibitor therapy, which suggests that elevated blood cholesterol confers a beneficial effect in anticancer immunotherapies. Additionally, the lipid paradox in autoimmunity demonstrates that lowered blood cholesterol is associated with a hyperinflammatory state, with levels returning to normal with anti-inflammatory treatment. This is a clear sign that cholesterol dysregulation is related to the immune response in conditions beyond cancer. We therefore consider how cholesterol modulating drugs, such as statins, could be used to bolster the anti-tumour immune response. While there is some promising data correlating improved CPI responses with statin use, there is no data exploring the use of statins in patients without hypercholesterolaemia. To understand whether the statins themselves are beneficial, this would be an interesting avenue for further study.

Finally, we consider additional potential adjuvant therapies that may confer benefit alongside immunotherapy. GLP-1RAs are a burgeoning class of drug used to treat type-2 diabetes and obesity and have been shown to have profound systemic anti-inflammatory effects. While this may seem anathema to CPI therapy, which relies on increasing T cell effector function, the data is conflicting. Mouse models have shown that in some cases, GLP1-RAs are a useful adjuvant treatment, while others (particularly neuroendocrine tumours) show the opposite. With a fast-increasing cohort of patients taking GLP-1RAs, the data around their effect on CPI treatment will likely emerge in the near future.

Alongside traditional drugs, we explore the potential benefits of nutrition modulation on CPI treatment effectiveness. In particular, fasting and ketogenic diets have demonstrated reprogramming of both tumour and immune cells and sensitisation of tumours to CPI therapies. Phenolic diterpenes have also shown a similar ability to reprogram immune and cancer cells and work synergistically alongside checkpoint blockade. Additionally, vitamins including Vitamin D, B12 and folate have all been implicated in CPI response, and their supplementation (for Vitamin D) or modulation (for B12 and folate) could be an effective strategy for improving treatment outcomes. Nutrition, whether through diet modulation or supplementation, can have a clear impact on both systemic metabolism and the anti-tumour immune response, and this represents a potential avenue for bolstering CPI efficacy using readily available resources.

While this review encapsulates the message that altering systemic metabolism can affect CPI treatment outcomes, there is clear gap in the existing literature for the inverse: an understanding of the impact of CPI treatment on systemic metabolism. It is our hope that going forward, this relationship is studied in greater detail, to unravel the mechanisms that underly successful CPI treatment, and give an insight into how we can make these revolutionary drugs effective for a greater number of patients.
